# FeGenie: A Comprehensive Tool for the Identification of Iron Genes and Iron Gene Neighborhoods in Genome and Metagenome Assemblies

**DOI:** 10.3389/fmicb.2020.00037

**Published:** 2020-01-31

**Authors:** Arkadiy I. Garber, Kenneth H. Nealson, Akihiro Okamoto, Sean M. McAllister, Clara S. Chan, Roman A. Barco, Nancy Merino

**Affiliations:** ^1^Department of Earth Sciences, University of Southern California, Los Angeles, CA, United States; ^2^Department of Earth Sciences, University of Delaware, Newark, DE, United States; ^3^International Center for Materials Nanoarchitectonics, National Institute for Materials Science, Tsukuba, Japan; ^4^School of Marine Science and Policy, University of Delaware, Newark, DE, United States; ^5^Earth-Life Science Institute, Tokyo Institute of Technology, Tokyo, Japan; ^6^Biosciences and Biotechnology Division, Lawrence Livermore National Laboratory, Livermore, CA, United States

**Keywords:** hidden Markov model (HMM) database, iron transport, iron storage, iron oxidation, iron reduction, iron gene regulation, magnetosome, siderophore

## Abstract

Iron is a micronutrient for nearly all life on Earth. It can be used as an electron donor and electron acceptor by iron-oxidizing and iron-reducing microorganisms and is used in a variety of biological processes, including photosynthesis and respiration. While it is the fourth most abundant metal in the Earth’s crust, iron is often limiting for growth in oxic environments because it is readily oxidized and precipitated. Much of our understanding of how microorganisms compete for and utilize iron is based on laboratory experiments. However, the advent of next-generation sequencing and surge in publicly available sequence data has made it possible to probe the structure and function of microbial communities in the environment. To bridge the gap between our understanding of iron acquisition, iron redox cycling, iron storage, and magnetosome formation in model microorganisms and the plethora of sequence data available from environmental studies, we have created a comprehensive database of hidden Markov models (HMMs) based on genes related to iron acquisition, storage, and reduction/oxidation in *Bacteria* and *Archaea*. Along with this database, we present FeGenie, a bioinformatics tool that accepts genome and metagenome assemblies as input and uses our comprehensive HMM database to annotate provided datasets with respect to iron-related genes and gene neighborhood. An important contribution of this tool is the efficient identification of genes involved in iron oxidation and dissimilatory iron reduction, which have been largely overlooked by standard annotation pipelines. We validated FeGenie against a selected set of 28 isolate genomes and showcase its utility in exploring iron genes present in 27 metagenomes, 4 isolate genomes from human oral biofilms, and 17 genomes from candidate organisms, including members of the candidate phyla radiation. We show that FeGenie accurately identifies iron genes in isolates. Furthermore, analysis of metagenomes using FeGenie demonstrates that the iron gene repertoire and abundance of each environment is correlated with iron richness. While this tool will not replace the reliability of culture-dependent analyses of microbial physiology, it provides reliable predictions derived from the most up-to-date genetic markers. FeGenie’s database will be maintained and continually updated as new genes are discovered. FeGenie is freely available: https://github.com/Arkadiy-Garber/FeGenie.

## Introduction

Iron is the fourth most abundant element in the Earth’s crust (Morgan and Anders, 1980), where it occurs primarily as ferrous [Fe(II)] or ferric [Fe(III)] iron. Under circumneutral pH and aerobic conditions, ferrous iron spontaneously oxidizes to its ferric form, which precipitates and settles out of solution becoming highly limiting to microbial life (Emerson, 2016). Nonetheless, microorganisms have evolved mechanisms to deal with this limitation, as evidenced by the variety of known enzymes responsible for iron scavenging (Barry and Challis, 2009), transport (Wyckoff et al., 2006; Toulza et al., 2012; Fillat, 2014; Lau et al., 2016), and storage (Smith, 2004; Rivera, 2017). While iron is limiting in many natural ecosystems, environments exist where iron concentrations are high enough to support communities of microorganisms capable of deriving energy from iron oxidation (Emerson and Moyer, 2002; Jewell et al., 2016). These environments can also be inhabited by microorganisms capable of using ferric iron, usually in the form of a mineral, as a terminal electron acceptor in electron transport chains (Gao et al., 2006; Emerson, 2009; Elliott et al., 2014; Quaiser et al., 2014). While various marker genes, based on the study of a few model organisms, have been inferred, relatively little is known about the genetics behind iron oxidation and reduction (He et al., 2017).

**FIGURE 1:**
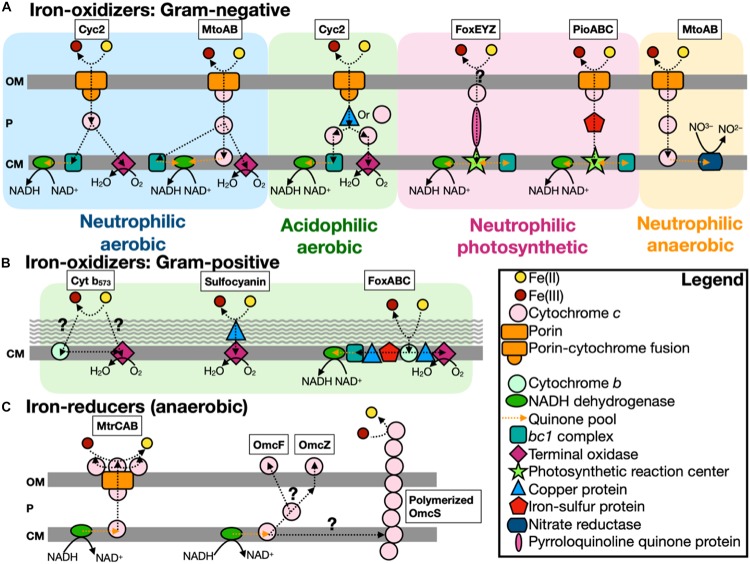
Scheme of known iron-oxidizers and iron-reducers. There are several different types of iron-oxidizers known, with more information on Gram-negative **(A)** bacteria compared to Gram-positive **(B)** bacteria (note: the acidophilic aerobic iron-oxidizers can use either a copper protein or cytochrome *c* to transfer electrons in the periplasm). **(C)** For iron-reducers, there are only two mechanisms known and under anaerobic conditions. The genes identified by FeGenie are in boxes above each type, with the exception of Cyt b573, which has yet to be confirmed for iron oxidation (White et al., 2016). FeGenie does not include pili and flavin-related genes since these genes are commonly associated with other functions/metabolisms. Modified from White et al. (2016) and Wang et al. (2019). OM, outer membrane; P, periplasm; and CM, cytoplasmic membrane.

**FIGURE 2:**
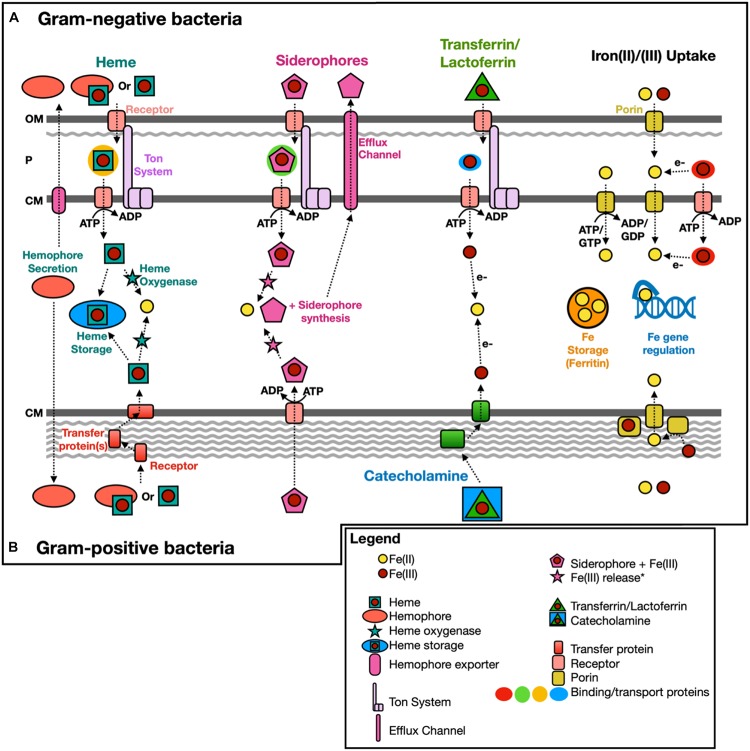
Scheme of known iron acquisition, storage, and regulation pathways. Gram-negative **(A)** and Gram-positive **(B)** bacteria have different mechanisms to uptake iron due to differences in the cell membrane structure. Iron(II)/(III) uptake can also be mediated extracellularly by redox cycling secondary metabolites, such as phenazine-1-carboxylic acid (Cornelis and Dingemans, 2013). OM, outer membrane; P, periplasm; and CM, cytoplasmic membrane. Modified from Anzaldi and Skaar (2010); Caza and Kronstad (2013), Contreras et al. (2014); Kranzler et al. (2014), Lau et al. (2016). *Fe(III) release from siderophores intracellularly could include Fe(III) reduction (e.g*., fpvG*, Ganne et al., 2017) or modification/hydrolysis of the siderophore (e.g., esterase, Brickman and McIntosh, 1992).

Microbial iron metabolisms (Figure 1) and acquisition/transport pathways (Figure 2) play significant roles across a wide range of environments. Indeed, the prevalence of iron as a necessary cofactor (Ayala-Castro et al., 2008) and the dependence of life on iron, with the exception of a group of homolactic bacteria (Pandey et al., 1994), suggests that life evolved in an iron-rich world. Moreover, the variety of microorganisms in the archaeal and the bacterial domains capable of using iron as an electron donor or acceptor (Nealson and Saffarini, 1994; Weiss et al., 2007; Hedrich et al., 2011; Ilbert and Bonnefoy, 2013; Fullerton et al., 2017) suggests that these metabolisms were either adopted very early in the history of life or benefited from horizontal gene acquisition. There are examples of organisms that are considered “iron-free” and do not appear to encode genes associated with iron homeostasis, such as *Borrelia burgdorferi* (Andrews et al., 2003) and *Treponema pallidum* (Posey and Gherardini, 2000). However, as pointed out by Andrews et al. (2003), since these *Bacteria* are intracellular parasites, their genomes are small (∼1 Mbp) and encode only a subset of genes required for bacterial growth and survival; reliance on host iron-dependent metabolic processes likely resulted in these parasites losing iron-associated genes.

Over the past few decades, almost three hundred genes involved in iron transport, metabolism, and transformation of iron and iron-containing minerals (e.g., magnetite, hematite, ferrihydrite, olivine, etc.) have been identified. Only a small proportion of these genes are thought to be involved in dissimilatory iron reduction and the energy-deriving process of iron oxidation. These are generally not annotated as such by established gene annotation pipelines, such as RAST (Overbeek et al., 2014), GhostKOALA (Kanehisa et al., 2016), MAPLE (Arai et al., 2018), and InterProScan (Quevillon et al., 2005). There are also no publicly available hidden Markov models (HMMs) for genes involved in iron oxidation and reduction, with the exception of *mtrB* (TIGR03509) and *mtrC* (TIGR03507), which have HMMs available within the TIGRFAMS HMM database. Moreover, many iron-related gene operons contain genes that are not exclusive to iron metabolism, but, nonetheless, within that operon, play an important role in acquiring or transporting iron (e.g., *asbC* in the siderophore synthesis gene operon *asbABCDEF* is annotated as an AMP-binding enzyme by the Pfam database). Herein, we make a publicly available set of HMMs based on current knowledge of iron acquisition, storage and respiratory oxidation/reduction mechanisms, and integrate that with HMMs based on all available genetic markers for iron acquisition, storage, and redox cycling in *Bacteria* and *Archaea*.

We present FeGenie, a new bioinformatics tool that comes with a curated and publicly available database of profile HMMs for enzymes involved in iron acquisition, storage, and redox-cycling in prokaryotes. FeGenie is available as a command-line tool, installed manually or *via* Conda configuration^1^. Users can submit genomes and metagenomes (in the form of contigs, amino acid gene sequences, or GenBank format files) for identification of known iron-related pathways. FeGenie consists of 208 protein families representing 12 iron-related functional categories (summarized in Table 1 and Supplementary Table S1). These functions are distributed across five overarching categories: iron acquisition/transport, iron storage, iron gene regulation, iron redox reactions, and magnetosome formation. HMMs were either manually constructed or taken from Pfam/TIGRFAMS. The advantage of using HMMs, as compared to local sequence alignments, is the rapid and sensitive identification of distantly related homologs to genes of interest (Eddy, 2004). This is particularly important in the analysis of large environmental datasets with uncultivated and/or novel microorganisms.

**TABLE 1:** Summary of iron-related protein families that are represented as pHMMs in FeGenie.

**Category**	**Function**	**Protein Families**
Iron acquisition	Iron(II)/(III) transport	Efe**U**OB^1^, FbpABC^2^, SfuABC^3^, YfuABC^4^, **FeoAB(C)**^5^, FutA1^6^, FutA2^6^, FutB^6^, FutC^6^, YfeABCD^7^
	Heme oxygenase	ChuS^8^, ChuZ^9^, HemO^10,11^, PigA^10,11^, Hem**RS**T**U**V^12^, HmoB^13^, HmuO^14^, HugZ^15^, HupZ^16^, Isd-LmHde^17^, IsdG^18^, IsdI^19^, MhuD^20^, **PhuS**^21^ (in PhuRSTUVW)
	Heme transport	Has**RA**DE(B)F^22^, HmuR**S**T**U**V^22^, **HmuY**^23^, **HmuY’**^23^, HutZ^24^, Hxu**C**BA^25^, **IsdX1**^26^, **IsdX2**^26^, Phu**RS**T**U**V**W**^21^, **Rv0203**^27^
	Transferrin/Lactoferrin	Tbp**A**B (Lbp**A**B)^28^, Sst**AB**CD^29^
	Siderophore synthesis	Acs**A**B**CD**EF^30^, AmoA^31^, AngR^32^, Asb**AB**CDEF^33^, DhbACEBF^34^, entD-**fepA**-fes-entF-fepEC**GDB**-entCEBA-ybdA^35^, IroD in Iro**N**BCDE^36^, IucABCD^37,38^, **IutA**^37,38^, MbtIJABCDEFGH^35^, LbtA^39^ (in LbtUABC), PchABCDREFGHI^35^, PvdQAPMNOFEDJIHLGS^40^, PvsABCDE^41^, VenB^42^, Vab genes in VabR-fur-vabGA-fur-VabCEBSFH-fur-fvtA-vabD^43^, Vib genes in VibB-vibEC-vibA-vibH-viuP**DG**C-vibD and viuAB-vibF^44–46^, RhbABCDEF-rhrA-rhtA^47^
	Siderophore transport	BesA^48^, CbrA**BC**D^49^, **TonB**-**ExbB**-**ExbD**^50^, Fat**A**B**CD**^51^, FecI**RA**B**CD**E^52^, FeuABC-yusV^53^, Fhu**A**CDB^54–56^, **FhuF**^54–56^, Fpt**A**BCX^57^, FpuA**B**^58^, FpuC^58^, FpuD^58^, FpvI**R**-**FpvA**-FpvGHJKCDEF^59^, FvtA in VabR-fur-vabGA-fur-VabCEBSFH-fur-fvtA-vabD^43^, HatCD**B**^37^, IroNBCDE^36^, LbtUABC^39^, PirA^60^, PiuA^60^, Pvu**A**BCDE^41^, Viu genes in VibB-vibEC-vibA-vibH-viuP**DG**C-vibD and viuAB-vibF^44–46^, **YfiZ**-yfhA^61^, YfiY^61^, YqjH^62^, ybdA and Fep genes in entD-**fepA**-fes-entF-fepEC**GDB**-entCEBA-ybdA^35^
Iron Gene regulation	Transcriptional regulation	**DtxR**^63^, FecR (in FecIRABCDE)^52^, **FeoC** in FeoAB(C)^5^, **Fur**^64^, **IdeR**^65^, YqjI^62^, RhrA in RhbABCDEF-rhrA-rhtA^47^
Iron oxidation and reduction	Iron oxidation	Cyc1^66,67^, Cyc2^66,67,68^, FoxABC^69^, FoxEYZ^70^, Sulfocyanin^71^, PioA**B**C^72^
	Probable iron oxidation and possible iron reduction	MtoA**B**^73^, Cyc2 (cluster 3)
	Dissimilatory iron reduction	CymA^74^, Mtr**C**A**B**^75^, OmcF^76^, OmcS^76^, OmcZ^76^, FmnA-dmkA-fmnB-pplA-ndh2-eetAB-dmkB^77^, DFE_0448-0451, DFE_0461-0465^78^
	Probable iron reduction	MtrCB, MtrAB, MtoAB-MtrC
Iron storage	Iron storage	**Bfr**^79^, **DpsA**^80^, **Ftn**^81^
Magnetosome-related	Magnetosome formation	MamABEKLMOPQI^82,83^ (Note: These genes are found in all known magnetotactic microorganisms, except for *mamL* which is found in magnetite-producing magnetotactic microorganisms^81^)

*Bolded and underlined HMMs are derived from Pfam or TIGRFAMs databases. Other HMMs were created by using select sequences. See Supplementary Table S1 for more information, including the corresponding Pfam or TIGRFAMs families and the sequences used to create the HMMs. ^1^Miethke et al., 2013, ^2^Adhikari et al., 1996, ^3^Angerer et al., 1990, ^4^Gong et al., 2001, ^5^Lau et al., 2016, ^6^Katoh et al., 2001, ^7^Bearden et al., 1998, ^8^Suits et al., 2006, ^9^Zhang et al., 2011, ^10^Friedman et al., 2003, ^11^Friedman et al., 2004, ^12^Schneider et al., 2006, ^13^Park et al., 2012, ^14^Matsui et al., 2005, ^15^Hu et al., 2011, ^16^Sachla et al., 2016, ^17^Duong et al., 2014, ^18^Reniere et al., 2010, ^19^Skaar et al., 2004, ^20^Graves et al., 2014, ^21^Ochsner et al., 2000, ^22^Tong and Guo, 2009, ^23^Wójtowicz et al., 2009, ^24^Liu X. et al., 2012, ^25^Morton et al., 2007, ^26^Honsa et al., 2014, ^27^Tullius et al., 2011, ^28^Gray-Owen et al., 1995, ^29^Morrissey et al., 2000, ^30^Carroll and Moore, 2018, ^31^Barghouthi et al., 1991, ^32^Wertheimer et al., 1999, ^33^Oves-Costales et al., 2007, ^34^May et al., 2001, ^35^Crosa and Walsh, 2002, ^36^Hantke et al., 2003, ^37^Suzuki et al., 2006, ^38^Martínez et al., 1994, ^39^Cianciotto, 2015, ^40^Lamont and Martin, 2003, ^41^Tanabe et al., 2003, ^42^Tan et al., 2014, ^43^Balado et al., 2008, ^44^Wyckoff et al., 2001, ^45^Keating et al., 2000, ^46^Wyckoff et al., 1999, ^47^Lynch et al., 2001, ^48^Miethke et al., 2006, ^49^Mahé et al., 1995, ^50^Garcia-Herrero et al., 2007, ^51^Lemos et al., 2010, ^52^Braun, 2003, ^53^Peuckert et al., 2011, ^54^Köster and Braun, 1989, ^55^Coulton et al., 1987, ^56^Braun et al., 2002, ^57^Youard et al., 2011, ^58^Dixon et al., 2012, ^59^Brillet et al., 2012, ^60^Moynie et al., 2017, ^61^Ollinger et al., 2006, ^62^Wang et al., 2011, ^63^Guedon and Helmann, 2003^,^^64^Escolar et al., 1998, ^65^Rodriguez et al., 2002, ^66^Castelle et al., 2008, ^67^Barco et al., 2015, ^68^McAllister et al., 2019, ^69^Bathe and Norris, 2007, ^70^Croal et al., 2007, ^71^Ilbert and Bonnefoy, 2013, ^72^Liu J. et al., 2012, ^73^Jiao and Newman, 2007, ^74^Castelle et al., 2015, ^75^Pitts et al., 2003, ^76^Santos et al., 2015, ^77^Light et al., 2018, ^78^Deng et al., 2018, ^79^Grossman et al., 1992, ^80^Grant et al., 1998, ^81^Andrews, 1998, ^82^Uebe and Schuler, 2016, ^83^Kolinko et al., 2016.*

To validate FeGenie, we tested the program against 28 microbial genomes (Supplementary Table S2) with established pathways for iron acquisition, iron oxidation, and iron reduction. These genomes are comprised of model organisms, including siderophore-producers, magnetotactic bacteria, iron-reducers, as well as known and suspected iron-oxidizers. We demonstrate that this tool efficiently identifies iron-related genes and potential operons present within selected representative genomes, accurately identifying iron oxidation and reduction genes in known and potential iron-oxidizers and iron-reducers, respectively. FeGenie was also used to analyze members of the recently discovered Candidate Phyla Radiation (CPR) (Brown et al., 2015) and other candidate taxa, as well as 27 publicly-available metagenomes, representative of a range of habitats that include iron-rich and iron-poor marine and terrestrial systems (Table 2). We present the results of these analyses and establish FeGenie as a straightforward and simple tool for the identification of iron-related pathways in genomes and metagenomes.

**TABLE 2:** Summary of metagenomes analyzed.

**Dataset**	**Environment description**	**NCBI Accession No.**	**Predicted ORFs**	**References**
Amazon River Plume (Station 3)	River/ocean mixing, intermediate salinity	SAMN02628402	377,266	Satinsky et al., 2017
Amazon River Plume (Station 10)	River/ocean mixing, low salinity	SAMN02628416	143,340	Satinsky et al., 2017
Amazon River Plume (Station 27)	River/ocean mixing, high salinity	SAMN02628424	278,301	Satinsky et al., 2017
The Cedars (BS5 2011)	Serpentinizing, alkaline groundwater (shallow source)	GCA_002583255.1	32,646	Suzuki et al., 2017
The Cedars (BS5 2012)	Serpentinizing, alkaline groundwater (shallow source)	GCA_002581825.1	50,323	Suzuki et al., 2017
The Cedars (GPS1 2011)	Serpentinizing, alkaline groundwater (deep source)	GCA_002581705.1	86,466	Suzuki et al., 2017
The Cedars (GPS1 2012)	Serpentinizing, alkaline groundwater (deep source)	GCA_002581605.1	78,321	Suzuki et al., 2017
Jinata Hot Springs	Iron-rich groundwater, mixed with seawater	PRJNA392119	992,695	Ward, 2017
Loihi Seamount (S1) (i.e., Syringe Sample)	Marine hydrothermal vent Fe microbial mat (surficial syringe sample)	SRR6114197	146,898	McAllister et al., 2019
Loihi Seamount (S6) (i.e., Scoop Sample 1)	Marine hydrothermal vent Fe microbial mat (bulk scoop sample)	Gp0295815	390,888	McAllister et al., 2019
Loihi Seamount (S19) (i.e., Scoop Sample 2)	Marine hydrothermal vent Fe microbial mat (bulk scoop sample)	Gp0295816	827,472	McAllister et al., 2019
Mid-Atlantic Ridge, Rainbow (664-BS3) (i.e., Syringe Sample 1)	Marine hydrothermal vent Fe microbial mat (surface syringe sample)	Gp0295819	414,137	McAllister et al., 2019
Mid-Atlantic Ridge, Rainbow (664-SC8) (i.e., Scoop Sample)	Marine hydrothermal vent Fe microbial mat (bulk scoop sample)	Gp0295820	597,486	McAllister et al., 2019
Mid-Atlantic Ridge, TAG (665-MMA12) (i.e., Syringe Sample 2)	Marine hydrothermal vent Fe microbial mat (surface syringe sample)	Gp0295821	255,314	McAllister et al., 2019
Mid-Atlantic Ridge, Snakepit (667-BS4) (i.e., Syringe Sample 3)	Marine hydrothermal vent Fe microbial mat (surface syringe sample)	Gp0295823	422,234	McAllister et al., 2019
Mariana Backarc, Urashima (801-BM1-B4, S7) (i.e., Scoop Sample)	Marine hydrothermal vent Fe microbial mat (surface syringe sample)	Gp0295817	365,851	McAllister et al., 2019
Arabian Sea metagenome (*Tara*)	Marine surface water	PRJNA391943	398,870	Tully et al., 2018
Chile/Peru Coast metagenome (*Tara*)	Marine surface water	PRJNA391943	375,779	Tully et al., 2018
East Africa Coast metagenome (*Tara*)	Marine surface water	PRJNA391943	464,070	Tully et al., 2018
Indian Ocean metagenome (*Tara*)	Marine surface water	PRJNA391943	178,873	Tully et al., 2018
Mediterranean metagenome (*Tara*)	Marine surface water	PRJNA391943	607,005	Tully et al., 2018
North Atlantic metagenome (*Tara*)	Marine surface water	PRJNA391943	673,120	Tully et al., 2018
North Pacific metagenome (*Tara*)	Marine surface water	PRJNA391943	601,358	Tully et al., 2018
Red Sea metagenome (*Tara*)	Marine surface water	PRJNA391943	331,387	Tully et al., 2018
South Atlantic metagenome (*Tara*)	Marine surface water	PRJNA391943	735,385	Tully et al., 2018
South Pacific metagenome (*Tara*)	Marine surface water	PRJNA391943	1,128,901	Tully et al., 2018
Rifle Aquifer	Terrestrial subsurface aquifer	Jewell et al., 2016 (Supplementary Data)	203,744	Jewell et al., 2016

*The list of 27 previously published metagenomes, representing a wide range of habitats from iron-rich to iron-poor marine and terrestrial systems. *Prodigal* v. 2.6.3 (Hyatt et al., 2010) was used to predict the number of open reading frames (ORFs) in each metagenome dataset. See section “Acquisition and assembly of environmental metagenomes” in the Materials and Methods for more detailed description and acquisition.*

## Materials and Methods

### Algorithm Overview

**FIGURE 3:**
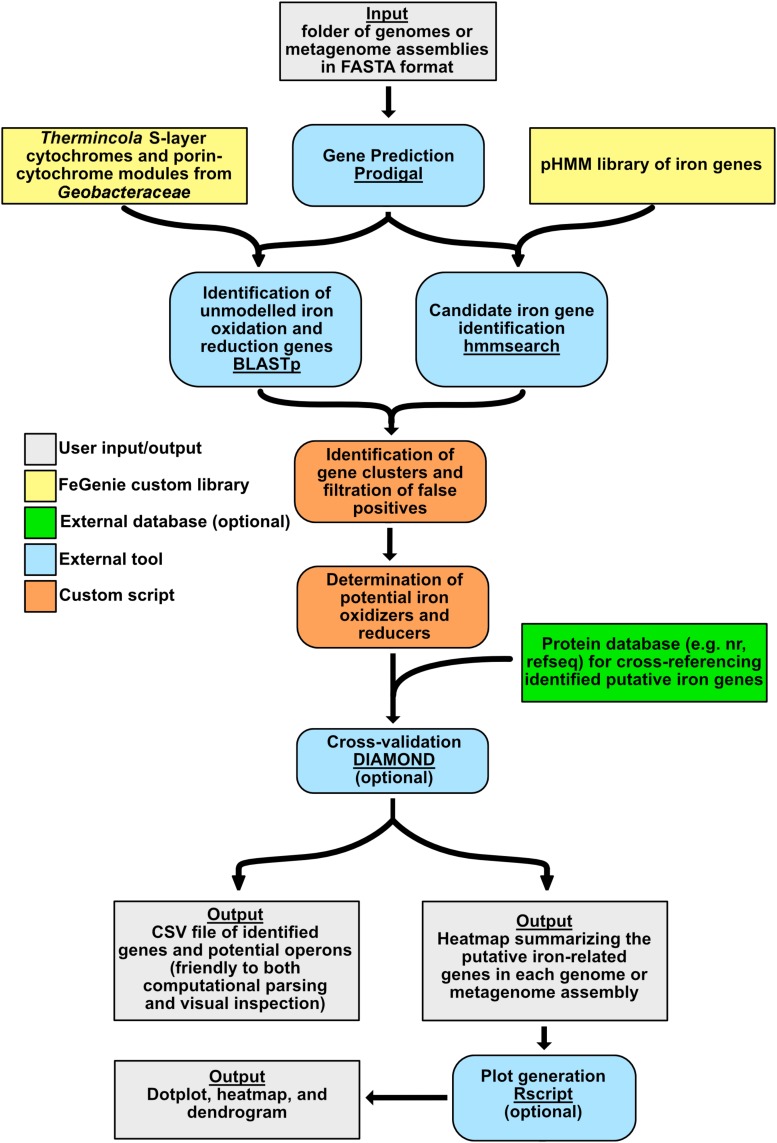
FeGenie algorithm overview. Color-coded to represent various aspects of the program, including external programs/dependencies, optional databases for cross-reference, and custom Python scripts.

FeGenie is implemented in Python 3, with three required dependencies: *HMMER* v. 3.2.1 (Johnson et al., 2010), *BLASTp* v. 2.7.1 (Madden, 2013), and *Prodigal* v. 2.6.3 (Hyatt et al., 2010). External installation of these dependencies is not required if FeGenie is configured using Conda^2^. There are two optional dependencies, which must be installed externally: *R* (R Core Team, 2013) and *Rscript* (R Core Team, 2013). R packages used in FeGenie include *argparse* (Davis, 2018), *ggplot2* (Wickham, 2009), *ggdendro* (de Vries and Ripley, 2016), *reshape* (Wickham, 2007), *reshape2* (Wickham, 2007), *grid* (R Core Team, 2013), *ggpubr* (Kassambara, 2017), *tidyverse* (Wickham, 2017), and *Pvclust* (Suzuki and Shimodaira, 2006); users need to install these packages independently using *Rscript* (detailed instructions on this are available within the FeGenie Wiki^3^). The overall workflow of FeGenie is outlined in Figure 3. User-provided input to this program includes a folder of genomes or metagenomes, which must all be in FASTA format, comprised of contigs or scaffolds. Users can also submit amino acid gene sequences in FASTA or GenBank format. First, *Prodigal* (Hyatt et al., 2010) is used to predict open-reading frames (ORFs). A custom library of profile HMMs (library described in section “HMM Development: Building and Calibrating HMMs”) is then queried against these ORFs using *hmmsearch* (Johnson et al., 2010), with custom bit score cutoffs for each HMM. Additionally, genes shown to be involved in dissimilatory iron reduction but lacking sufficient homologs in public repositories (precluding us from building reliable HMMs) are queried against the user-provided dataset using *BLASTp* (Madden, 2013) with a default e-value cutoff of 1E-10. These genes include the S-layer proteins implicated in iron reduction in *Thermincola potens* JR (Carlson et al., 2012), as well as porin-cytochrome encoding operons implicated in iron reduction in *Geobacter* spp. (Shi et al., 2014). The results of *hmmsearch* (Johnson et al., 2010) and *BLAST* (Madden, 2013) are then analyzed and candidate gene neighborhoods identified. Potential for dissimilatory iron oxidation and reduction is determined based on a set of rules that are summarized in Supplementary Table S3. Even though the sensitivity of each HMM has been calibrated against NCBI’s nr database (see section “HMM Development: Building and Calibrating HMMs*”* for details on the calibration process), we recommend that users take advantage of an optional cross-validation feature of the program that allows users to search each FeGenie-identified putative iron gene against a user chosen database of reference proteins (e.g., NCBI’s nr, RefSeq). Based on these analyses, FeGenie outputs the following files:

1. CSV file summarizing all identified putative iron-related genes, their functional category, bit score (shown in the context of the calibrated bit score cutoff of the matching HMM), number of canonical heme-binding motifs, amino acid sequence, and closest homolog to a user-provided database (optional; e.g., NCBI nr database).
2. Heatmap summary comparing the number of genes identified from each iron-related category across the analyzed genomes/metagenomes.
3. Three plots created with Rscript (optional): (1) Dendrogram showing the dissimilarity (based on iron-gene distributions) between provided genomes or assemblies, (2) scaled heatmap based on the relative distribution of iron-related genes across genomes/metagenomes, and (3) dot plot showing the relative abundance of iron genes across genomes. Using the R package *Pvclust* (Suzuki and Shimodaira, 2006), the dendrogram is produced using Ward’s method and Euclidian distance metric to hierarchically cluster the data with bootstrapped probability values for each cluster. This will allow users to assess the uncertainty in clustering, given that the analyzed genomes and metagenomes may not necessarily be derived from extremely different environments and iron/redox regimes.

### HMM Development: Building and Calibrating HMMs

Collection of iron-related protein sequences occurred between May 2018 and August 2019. FeGenie’s HMM library includes genes associated with iron acquisition from the environment, iron storage, magnetosome formation, and iron redox-cycling. This tool does not include genes related to downstream iron utilization pathways, such as a heme and iron-sulfur cluster synthesis. Moreover, FeGenie’s HMM library does not include genes associated with small regulatory RNAs. Sequences corresponding to proteins whose functions have been characterized in the literature were downloaded from reviewed sequences on UniProtKB (The UniProt Consortium, 2017) or NCBI, excluding proteins that were already represented by Pfam families (Finn et al., 2016) (Supplementary Table S1). To expand the diversity of each of the collected proteins, those sequences were then used as queries in a *BLASTp* v.2.6.0 (Madden, 2013) search against NCBI’s RefSeq (Release 89) database (Pruitt et al., 2007), with a minimum amino acid identity cutoff of 35% (Rost, 1999) over at least 70% of the query length. These search results were then de-replicated so that each seed sequence is represented by a unique set of non-overlapping BLAST hits. Using *MMseqs2* (Steinegger and Söding, 2017), each seed sequence and its set of BLAST hits were then collapsed with a 70% amino acid identity cutoff to remove overrepresented protein sequences, which would otherwise create biases in resulting HMMs. Each collapsed set of sequences was then aligned using *Muscle* v.3.8.31 (Edgar, 2004) and each alignment was manually inspected and curated. These curated alignments were then used as seeds for the generation of HMMs using the *hmmbuild* command from *HMMER* (Johnson et al., 2010). To calibrate appropriate bit score cutoffs for each HMM in the HMM library, each HMM was queried against NCBI’s nr database (Pruitt et al., 2007) using *hmmsearch*. By manually inspecting each *hmmsearch* result, we identified bit score cutoffs that optimally delineated between true and false positives among hits from nr. Thus, each HMM in the FeGenie library received its own custom bit score cutoff. This library represents the most comprehensive set of proteins associated with iron metabolisms and pathways available at the time of collection. This database will be updated as new genes relevant to iron are discovered.

### HMM Development: Iron Oxidation/Reduction

For determination of iron oxidation potential, we included the candidate iron oxidase from acidophilic and neutrophilic iron-oxidizing bacteria, Cyc2 (Barco et al., 2015). As shown by McAllister et al. (2019), Cyc2 is represented by three phylogenetically distinct clusters; thus, we constructed three different HMMs, specific to each cluster. Cluster 1 includes sequences from most known, well-established neutrophilic iron-oxidizers but is yet to be genetically or biochemically verified as an iron oxidase. Clusters 2 and 3 include sequences from acidophilic iron-oxidizing bacteria, including two homologs that have been biochemically verified to catalyze the oxidation of iron: Cyc2 from *Acidithiobacillus ferrooxidans* (Castelle et al., 2008) and Cyt572 from *Leptospirillum rubarum* (Jeans et al., 2008).

FeGenie also includes MtoA as a possible, but as yet unconfirmed, indicator for iron oxidation potential (Liu J. et al., 2012). The function of MtoA is unclear since it is homologous to the iron-reducing enzyme, MtrA, of *Shewanella oneidensis* MR-1, but nonetheless it is proposed to be involved in iron oxidation by Liu J. et al. (2012), even though there is a lack of supporting gene expression data. Indeed, MtoA has been shown to rescue Δ*mtrA* mutants of MR-1, partially recovering the ability to reduce ferric iron (Liu J. et al., 2012). Nonetheless, phylogenetic analysis shows a separation between the *mtrA* genes utilized by known iron-reducing bacteria (particularly within the *Alteromonadaceae* and *Vibrionaceae* families), and *mtoA* homologs encoded by known and suspected iron-oxidizing bacteria (Garber, 2018), including members of the *Gallionellaceae* (Supplementary Figure S1). Thus, two separate HMMs were constructed, one for MtrA homologs encoded by known iron-reducers and one for MtoA homologs encoded by known and suspected iron-oxidizers. The MtoA HMM includes PioA, which has been genetically (Jiao and Newman, 2007) and experimentally (Gupta et al., 2019) verified to be necessary for iron oxidation in *Rhodopseudomonas palustris* TIE-1. Moreover, the *mtrA*-encoding operon in iron-reducing bacteria typically encodes *mtrC*, an outer-membrane cytochrome thought to participate in dissimilatory iron reduction (Lower et al., 2007). MtrC is not encoded by iron-oxidizing bacteria (Shi et al., 2014), supporting its use as an additional indicator for iron-reducing potential. In light of these ambiguities in the function of MtoA, identification of MtoAB by FeGenie is treated with caution as a potential iron oxidase/reductase. Other HMMs used for determination of iron oxidation potential include genes from iron-oxidizing *Archaea*: sulfocyanin (Castelle et al., 2015), *foxABC* (Bathe and Norris, 2007), and *foxEYZ* (Croal et al., 2007).

Determination of iron reduction potential is dependent on the identification of homologs to various porin-cytochrome operons, including *mtrCAB* (Pitts et al., 2003), as well as two operons from *Desulfovibrio ferrophilus* (Deng et al., 2018), various porin-cytochrome operons identified in *Geobacteraceae* (Shi et al., 2014), and genes encoding S-layer-associated proteins implicated in iron reduction in *Thermincola potens* JR (Carlson et al., 2012). Additionally, we included the flavin-dependent operon that was implicated in iron reduction in *Listeria monocytogenes* (Light et al., 2018).

Seed sequences for MtrA, MtoA, and Cyc2 were manually curated, aligned using *Muscle*, and used for the building of HMMs. Due to the highly divergent nature of the porin domain in Cyc2, identification of Cyc2 is dependent upon the presence of a heme-binding motif and length of at least 375 amino acids, which is considered long enough to encode an outer membrane porin (Tamm et al., 2004).

### HMM Development: Siderophore Synthesis

FeGenie can also be used to identify siderophore synthesis genes and potential operons. Siderophores are microbially produced products (500–1200 Da) that have a preference for binding ferric iron (up to 10^–53^ M) (Ehrlich and Newman, 2008), enabling microorganisms to obtain this largely insoluble iron form. There are over 500 identified siderophores, categorized as catecholates, hydroxamates, or hydroxycarboxylic acids (Kadi and Challis, 2009). Microorganisms can synthesize siderophores *via* the NRPS (non-ribosomal peptide synthetase) or NIS (NRPS-independent siderophore) pathways (Carroll and Moore, 2018). The NRPSs are megaenzymes that consist of modular domains (adenylation, thiolation, and condensation domains) to incorporate and sequentially link amino acids, keto acids, fatty acids, or hydroxy acids (Gulick, 2017). The NRPSs are highly selective and predictable based on the product produced, and FeGenie will identify these putative siderophore synthesis genes based on the genomic proximity of each identified gene (Table 1). In contrast, the NIS pathway consists of multiple enzymes that each have a single role in the production of a siderophore, such as aerobactin, which was the first siderophore discovered to be synthesized by this pathway (Kadi and Challis, 2009). The operon involved in aerobactin biosynthesis is *iucABCD*, and homologs of the genes *iucA* and *iucC* (which are included in FeGenie) are indicators of siderophore production *via* the NIS pathway (Carroll and Moore, 2018). The HMM library that represents siderophore synthesis consists of HMMs derived from the Pfam database, as well as those constructed here (Table 1). Because many different siderophore synthesis pathways share homologous genes, we developed HMMs that were sensitive to the entirety of each gene family, rather than for each individual siderophore. Supplementary Data Sheet S1 summarizes the gene families from which HMMs were built and includes gene families for siderophore export, iron uptake and transport, and heme degradation. Although FeGenie cannot predict the exact siderophore produced, FeGenie enables users to identify putative (and potentially novel) siderophore synthesis operons, which can then be confirmed by external programs, such as *antiSMASH* (Weber et al., 2015), a bioinformatics tool to identify biosynthetic gene clusters.

### HMM Development: Siderophore and Heme Transport

Similar to siderophore synthesis, transport genes for siderophores, heme/hemophores, and iron from transferrin/lactoferrin are represented by HMMs specific to gene families. HMMs used by FeGenie to infer siderophore and heme transport include both custom-made and Pfam models (Table 1 and Supplementary Table S1). In Gram-positive bacteria, siderophores are delivered to an ATP-binding cassette (ABC) importer from a receptor protein (Brown and Holden, 2002) while hemes, hemophores, and iron from transferrin and lactoferrin are delivered via a receptor protein and a series of cell-wall chaperone proteins (Contreras et al., 2014). In comparison, for Gram-negative bacteria, the Ton system (TonB-ExbB-ExbD protein complex) is the commonly used transport mechanism located in the cytoplasmic membrane (Figure 2) (Krewulak and Vogel, 2011; Contreras et al., 2014). Because the Ton system can uptake other metabolites (e.g., vitamin B12), the identification of this transport pathway suggests only the *potential* for the transport of siderophores, hemes, and iron from transferrin/lactoferrin; it is the sole system known to transport these iron-bearing molecules, thus far, in Gram-negative bacteria (Faraldo-Gómez and Sansom, 2003; Caza and Kronstad, 2013). For example, *Pseudomonas aeruginosa* PAO1 encodes 34 different TonB-dependent receptors, including PiuA and PirA (Luscher et al., 2018). While this diversity of TonB-dependent receptors reflects, in part, an ability to uptake multiple types of siderophores, it also indicates that these receptors are also likely utilized for purposes outside of iron metabolism. Thus, it is possible that FeGenie overestimates the potential for the transport of iron-bearing compounds. In light of this potential for overestimation, caused by ambiguity related to the substrate targeted by TonB-dependent transport systems, we urge users to further investigate identified TonB-dependent receptors and not immediately interpret their presence as evidence of transport of siderophores, hemes, and/or iron from transferrin/lactoferrin.

### HMM Development: Iron Uptake

FeGenie also features a set of genes implicated in the transport of ferrous and ferric iron ions. Some examples of these include *futA1* and *futA2* (Katoh et al., 2001), which bind both ferrous and ferric iron (Kranzler et al., 2014), although there is preference for Fe(II) (Koropatkin et al., 2007). Some iron transporters may also work in conjunction with the transport of heme, siderophore, or iron from transferrin/lactoferrin, such as the iron transport operon *EfeUOB*. Other genetic markers for iron transport encompassed by FeGenie’s HMM library include *feoABC* (Lau et al., 2016), *fbpABC* (Adhikari et al., 1996), and others listed in Table 1 and Supplementary Table S1.

### HMM Development: Heme Transport and Lysis

Heme oxygenase and transport genes define another strategy that microorganisms, especially pathogens, use to obtain iron from their environment. In particular, heme oxygenases enable pathogens to obtain iron from a host through oxidative cleavage of heme, thereby releasing iron (Wilks and Heinzl, 2014). Heme oxygenases are categorized into two groups: (1) “canonical” heme oxygenases (HmuO, PigA, and HemO), which degrade heme to biliverdin and carbon monoxide, and (2) “non-canonical” heme oxygenases (IsdG, IsdI, MhuD, and Isd-LmHde), which degrade heme to products like staphylobilin (IsdG and IsdI) and mycobilin (MhuD) (Wilks and Heinzl, 2014). All these heme oxygenase genes are included in FeGenie’s HMM library. Similarly, orthologs to known heme transport genes are also identified by FeGenie, including the five bacterial heme transport systems (Contreras et al., 2014): IsdX1, IsdX2, HasA, HxuA, and Rv0203.

### HMM Development: Regulation

Regulation of iron uptake and storage is an important aspect of iron homeostasis. Microorganisms often reside in ever-changing conditions and must sense and respond to their outside environment with respect to iron transport. To this end, genes encoding transcriptional regulators modulate the expression of various genes relevant to iron acquisition. We included these genes in FeGenie’s HMM library. These transcriptional regulators include FeoC, which functions as an iron sensor and repressor of the *feo* operon (Lau et al., 2016). Another important transcriptional regulator is ferric uptake regulator Fur, which binds ferrous iron and represses siderophore synthesis and iron uptake. Fur is also thought to control expression of genes involved in reactive oxygen species neutralization (Troxell and Hassan, 2013). Other regulators represented in FeGenie’s HMM library include PchR (Heinrichs and Poole, 1996), DtxR (Brune et al., 2006), and YqjH (Wang et al., 2011).

Extracytoplasmic-function (ECF) sigma factors are sensitive to signals from outside of the cell, and they bind and recruit RNA-polymerase to specific regions of the genome (Brooks and Buchanan, 2008). Two such ECF sigma factors, PvdS and FpvI, are included in FeGenie’s HMM library. PvdS controls expression of genes for pyoverdine biosynthesis, while FpvI controls expression of a TonB-dependent siderophore receptor (Reinhart and Oglesby-Sherrouse, 2016). In addition, we also included the FecR regulatory protein, which signals an (ECF) sigma factor to promote the expression of genes responsible for ferric citrate transport (Stiefel et al., 2001).

### Acquisition of Representative Genomes From RefSeq and Candidate Taxa

Genome sequences were downloaded from the NCBI RefSeq and GenBank database (Pruitt et al., 2007) on November 4, 2017. Genomes from the Candidate Phyla Radiation and other candidate taxa were obtained using the NCBI accession IDs found in Hug et al. (2016). All NCBI accessions are listed in Supplementary Table S2, as well as Supplementary Data Sheets S2, S3, S10, S11.

### Acquisition and Assembly of Environmental Metagenomes

• *Loihi Seamount, Mid-Atlantic Ridge, and Mariana Backarc Iron microbial mats*: Eight iron mat metagenomes, three from Loihi Seamount, four from the Mid-Atlantic Ridge, and one from the Mariana Backarc, were sequenced and assembled (details in McAllister et al., 2019). Syringe samples represent active samples from the edge of iron mats. Scoop and slurp samples represent bulk samples, which include deeper mat material. Assembly data available from JGI Sequence Project IDs Gp0295814-Gp0295821 and Gp0295823.
• *The Cedars, a terrestrial serpentinite-hosted system*: Metagenome assemblies were downloaded from the NCBI GenBank database (BioProject Accession ID: PRJDB2971): GCA_002581605.1 (GPS1 2012), GCA_002581705.1 (GPS1 2011), GCA_002581825.1 (BS5 2012), and GCA_002583255.1 (BS5 2011) (Suzuki et al., 2017). GPS1 (Grotto Pool Springs) is sourced by deep groundwater while BS5 (Barnes Springs 5) is sourced by ∼15% deep groundwater and ∼85% shallow groundwater. Both environments host highly alkaline and highly reducing waters. Two samples were collected from each spring and represent temporal duplicates taken approximately 1 year apart. These metagenomes were processed as described in Suzuki et al. (2017).
• *Amazon River plume estuary*: Raw metagenome reads were downloaded from NCBI’s Sequence Read Archive (SRA) corresponding to BioSamples SAMN02628402 (Station 3), SAMN02628424 (Station 27), and SAMN02628416 (Station 10); these correspond to samples taken along a salinity gradient formed as the Amazon River flows into the Atlantic Ocean (Satinsky et al., 2017). Station 10 represents water samples taken nearest to the source of river water, and Station 27 represents the sample taken furthest away from the river. Raw reads were quality trimmed using *Trimmomatic* v.0.36 (Bolger et al., 2014) with a sliding window of 4 base pairs (bp) and minimum average quality threshold of 15 (phred33) within that window; reads shorter than 36 bp were discarded. *SPAdes* v.3.10 (Bankevich et al., 2012) with the ‘–meta’ flag (Nurk et al., 2017) and default k-mers was used for assembly of high-quality reads into contigs.
• *Jinata Hot Springs*: This metagenome assembly was provided by Dr. Lewis Ward and processed as described by Ward et al. (2019). The assembly is located in the NCBI database under accession PRJNA392119. Raw metagenome data are represented by accession numbers SRX4741377-SRX4741380. This ecosystem represents a hot spring where low-oxygen and iron-rich fresh groundwater mixes with oxic and iron-deplete ocean water.
• *Rifle Aquifer*: ORFs from the assembled Rifle Aquifer metagenome were downloaded from the supplemental dataset published by Jewell et al. (2016).
• *Tara Oceans*: Assembled and published contigs corresponding to the fraction that was binned into draft genomes were originally processed and analyzed by Tully et al. (2018) and downloaded from Figshare^4^. This dataset represents a globally distributed set of marine metagenomes collected from the sunlit portion of the water column. The global distribution is defined by the Longhurst geographical provinces.

## Results and Discussion

### Validation of FeGenie Against Isolate Genomes

**FIGURE 4:**
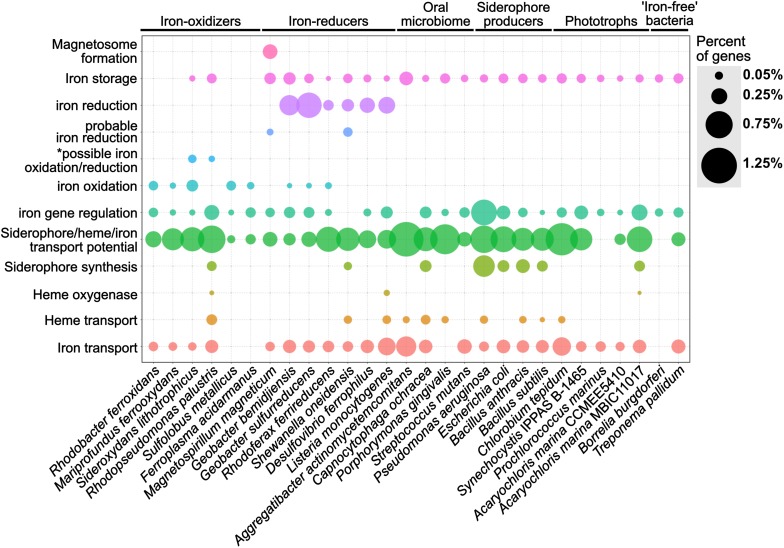
Dot plot showing the relative abundance of different iron gene categories within 28 representative isolate genomes. The isolate genomes were selected as model microorganisms to demonstrate the accuracy of FeGenie for identifying genes involved in iron oxidation and reduction, iron transport (including siderophores and heme), iron storage, and iron gene regulation. The genomes were obtained from the NCBI RefSeq and GenBank databases and analyzed by FeGenie. The size of each dot reflects the number of genes identified for each category and normalized to the number of protein-coding genes predicted within each genome. *This category is reserved for genes related to the mtoAB/pioAB gene family.

We validated FeGenie by showing that it accurately identifies and classifies iron-related genes in representative organisms known to encode them. A total of 574 representative genomes from RefSeq were analyzed, and these results are provided in Supplementary Data Sheets S2, S3. Here, we present the results from a select set of 28 genomes (Supplementary Table S2 and Supplementary Data Sheets S4, S5), including known iron-oxidizers (e.g., *Mariprofundus ferrooxydans* PV-1 and *Rhodopseudomonas palustris* TIE-1), iron-reducers (e.g., *Shewanella oneidensis* MR-1 and *Geobacter sulfurreducens* PCA), magnetotactic bacteria (*Magnetospirillum magneticum* AMB-1), siderophore synthesis and uptake model microorganisms (e.g., *Bacillus anthracis* and *Pseudomonas aeruginosa*), and others (as listed in Supplementary Table S1). These genomes were chosen to showcase FeGenie’s capacity to detect key genes relevant to the microbial iron-cycle (Figure 4).

Putative iron oxidation genes were detected in iron-oxidizing bacteria, including *Sideroxydans lithotrophicus* ES-1 (Emerson et al., 2013), *Rhodobacter ferrooxidans* SW2 (Croal et al., 2007), *Mariprofundus ferrooxydans* PV-1 (Emerson et al., 2007), *Rhodopseudomonas palustris* TIE-1 (Jiao et al., 2005), as well as in *Archaea*, including *Sulfolobus metallicus* (Bathe and Norris, 2007) and *Ferroplasma acidarmanus* (Golyshina et al., 2000). *S. lithotrophicus* is a known iron-oxidizer and was found to encode *mtoAB* (Liu J. et al., 2012) and three copies of *cyc2* within its genome (Emerson et al., 2013). Since *mtoAB* are homologous to genes also implicated in iron reduction (*mtrAB*), FeGenie classified these genes as potentially related to iron oxidation or iron reduction (i.e., the “possible iron oxidation/reduction” category).

FeGenie accurately identified iron-reduction genes and operons in known iron-reducing bacteria. For example, *Shewanella oneidensis* MR-1, a model organism for iron reduction, was found to encode both copies of its porin-cytochrome module: *mtrCAB* and *mtrDEF* (*mtrDEF* is homologous to *mtrCAB* and was identified as such by FeGenie). Additionally, FeGenie identified two more operons that each encode only *mtrAB*, which FeGenie categorizes as “probable iron reduction” due to the lack of *mtrC*. Interestingly, within the *mtrCABDEF* operon, FeGenie also identified the ferrous iron transport genes *feoAB*, which could be involved in the uptake of ferrous iron that is generated during iron reduction. This same operon also encodes a catalase (not included in FeGenie), which is a heme-containing protein that deals with oxidative stress and may potentially be expressed together with the iron-reduction genes to deal with the oxidative stress of high intracellular iron concentrations (Touati, 2000), likely resulting from dissimilatory iron reduction.

Some of the identified iron-reducers, for example, *R. ferrireducens* (Finneran et al., 2003), *G. sulfurreducens* (Lovley and Phillips, 1988) and *G. bemidjiensis*, also encode the cluster 3 *cyc2*, which FeGenie uses as a marker for iron oxidation. This gene has been confirmed as an iron-oxidase in *Acidithiobacillus ferrooxidans* (Castelle et al., 2008) and is also encoded by neutrophilic, obligate iron-oxidizers (Barco et al., 2015). We note that only one of these cluster 3-affiliated Cyc2 homologs, Cyt572, has been biochemically characterized and determined to have iron oxidase activity (Jeans et al., 2008). It is worth noting that *Geobacter metallireducens* has been previously shown to oxidize iron in a biological process known as nitrate-dependent iron oxidation (Weber et al., 2006) and does have a *cyc2* gene. Our results indicate that there are other *Geobacter* spp. that could also be involved in iron oxidation (either aerobically or anaerobically).

*Magnetospirillum magneticum* AMB-1, a known magnetotactic bacterium (Matsunaga et al., 2005), was positive for magnetosome formation genes. *M. magneticum* AMB-1 also encodes *mtrAB* which FeGenie uses as a marker for “probable iron reduction.” *M. magneticum* AMB-1 lacks the outer-membrane cytochrome (MtrC) that is always found within the *mtrCAB* operon of iron-reducing bacteria (Richardson et al., 2012; White et al., 2016). However, experimental evidence demonstrated that AMB-1 is an iron-reducing bacterium (Matsunaga et al., 2005). Without any other candidate iron reductases in AMB-1’s genome, this indicates that MtrAB may be utilized in iron reduction without the outer-membrane component.

FeGenie was also used to identify iron acquisition and transport genes in model microorganisms, including siderophore transport and synthesis genes, heme transport and oxygenases, and Fe(II)/Fe(III) transport. It is worth noting that in these organisms not linked to respiratory iron oxidation or dissimilatory iron reduction, FeGenie did not identify genes related to these metabolisms. In *Escherichia coli* and *Bacillus subtilis*, FeGenie identified three genes that are necessary for the uptake of iron, *efeUOB* (Cao et al., 2007), in addition to other iron transport genes (Supplementary Data Sheet S4). Iron transport potential was also identified in nearly every genome analyzed (including the CPR, discussed more in the section “*Case Study: Iron-related genes encoded within the Candidate Phyla Radiation and other Candidate Bacteria and Archaea*”). This is expected, given that iron is a necessary micronutrient for the vast majority of life. As an example of FeGenie’s capability to identify the siderophore gene families, we will focus on siderophore synthesis by *Bacillus anthracis*. *B. anthracis* is known to produce anthrabactin (*bacACEBF*) and petrobactin (*asbABCDEF*) (Oves-Costales et al., 2007). Both operons were correctly identified by FeGenie (Supplementary Data Sheet S5). Since the ORFs from each operon were annotated according to the gene family that each gene belongs to (Supplementary Data Sheet S1), users can cross-validate these genes with Supplementary Data Sheet S1 and confirm their identity through external pipelines. Further confirmation of these two operons by *antiSMASH* (Weber et al., 2015) (Supplementary Table S4) demonstrates the utility of FeGenie to identify siderophore synthesis gene operons.

Genes involved in heme transport and lysis were also identified in some of the model organisms. For example, in *Pseudomonas aeruginosa* PAO1, FeGenie identified *hasA* downstream to a TonB-dependent heme receptor. The rest of the *hasA* operon, however, was identified as part of the siderophore transport pathway. This is because some of the genes in the heme-transport operon *hasRADEF* are related to siderophore transport genes. This ambiguity in function demonstrates the weakness of FeGenie (and culture-independent, database-based approaches in general) and underscores the need to compare all identified putative iron-related genes against NCBI’s nr or RefSeq databases to see the annotations associated with the closest homologs available in public repositories. This step will add additional confidence that a gene identified as iron-related is indeed so, based on its closest known annotated homolog. Moreover, we stress the ambiguity presented by identification of TonB-dependent receptors. Although the TonB-dependent transport system is a commonly used mechanism in Gram-negative bacteria (Krewulak and Vogel, 2011; Contreras et al., 2014), as discussed in “Materials and Methods”, this family of proteins could also be utilized for transport of a wide variety of substrates, many of which are irrelevant to iron homeostasis.

FeGenie identified iron-relevant genes encoded by five phototrophs, *Chlorobium tepidum* TLS, *Synechocystis* IPPAS B-1465, *Prochlorococcus marinus*, and two strains of *Acaryochloris marinus*. As expected, the five analyzed phototrophs do not show genetic potential for iron oxidation or reduction. Generally, a higher number of genes related to iron and siderophore transport were identified in the anaerobic green-sulfur photoautotroph *C. tepidum* TLS, as compared to the freshwater and marine phototrophs, *Synechocystis* and *Prochlorococcus*, respectively. This may be due to the fact that *C. tepidum* performs anoxygenic photosynthesis in anaerobic, sulfide-rich niches (Eisen et al., 2002), which are often devoid of soluble iron. The lower iron conditions encountered by *C. tepidum* may necessitate higher genetic potential for iron acquisition. Interestingly, the open-ocean cyanobacterium *P. marinus* was not found to encode any genes for transport or synthesis of siderophores. Genes for heme transport or lysis were also not found in this genome. Indeed, *P. marinus* is known for its ability to subsist in low iron regimes, not through increasing its iron income but through lowering its iron expenditures (Partensky et al., 1999; Rusch et al., 2010). Nonetheless, *P. marinus* seems to encode genes involved in the storage (ferritin) and transport (*yfeAB*) of iron, and these gene were identified by FeGenie.

Using FeGenie, we compared iron gene inventories of two strains of the cyanobacterium *Acaryochloris marina*, MBIC11017 and CCMEE 5410. *Acaryochloris marina* are unique in that they use chlorophyll *d* to capture far-red light during photosynthesis (Swingley et al., 2008), a strategy that may have offered a competitive edge over other cyanobacteria, and led to genome expansion and accumulation of an unusually large number of gene duplicates (Swingley et al., 2008). FeGenie results demonstrate that strain MBIC11017 encodes more genes associated with iron acquisition via siderophore synthesis, iron/siderophore transport, and heme lysis. This is consistent with the isolation of MBIC11017 from a habitat that is more iron-deplete than the one from which CCMEE 5410 was isolated (Miller et al., 2011). Moreover, Miller et al. (2011) have reported a large number of gene duplicates in strain MBIC11017 that are predicted to be involved in iron acquisition. The duplication of genes involved in iron acquisition may be a strategy used for adaptation to a low-iron niche via increased gene dosage (Gallagher and Miller, 2018). The detection of these genomic differences by FeGenie further demonstrates its utility in genomic studies.

Regulators of iron genes were also detected in nearly all analyzed isolate genomes, with the exception of *Shewanella oneidensis* MR-1 and *Aggregatibacter actinomycetemcomitans*. Iron gene regulators are often found within operons encoding iron-related genes. For example, the *feo* operon, in addition to the transporters *feoA* and *feoB*, often encodes the regulator *feoC*. However, in some cases, instead of *feoC*, *feoAB* are in the same operon as *fur* (e.g., *Geobacter bemidjiensis*) or the iron efflux gene *feoE* (Bennett et al., 2015). Two iron-dependent repressors were also identified adjacent to a gene encoding the iron reduction protein OmcF in *Geobacter bemidjiensis*, further suggesting, as in *S. oneidensis*, that dissimilatory iron reduction could be linked to iron uptake. *P. aeruginosa* appears to encode the greatest number of iron gene regulators, such as PchR and Fur, as well as ECF sigma factors FpvI and PvdS, many of which are encoded in close proximity to genes relevant to siderophore synthesis and transport.

FeGenie was also used to analyze the genomes of two intracellular pathogens, which are considered “iron-free” organisms due to an apparent lack of genes associated with iron acquisition, storage, and utilization. FeGenie identified only a few potential iron-related genes. Within the genome of *Borrelia burgdorferi*, FeGenie identified a *fur*-family gene that encodes BosR, a zinc-dependent transcriptional regulator (Boylan et al., 2003; Katona et al., 2004), and a ferritin-family gene that encodes BicA, an iron and copper-binding protein that is thought to detoxify cells from iron and copper (Wang P. et al., 2012). BicA is part of a broader Dps (DNA-binding protein from starved bacteria) family of proteins. It is thought that *bicA* expression is regulated by BosR (Boylan et al., 2003). In *Treponema pallidum*, FeGenie identified three loci corresponding to candidate iron genes. In one locus, *T. pallidum* encodes a Dps-family protein, which may function to accumulate iron. FeGenie also identified two genes potentially involved in the transport of siderophores, hemes, or iron from transferrin/lactoferrin. Indeed, *T. pallidum* has been reported to bind host lactoferrin and transferrin (Alderete et al., 1988), and may do so using the permease and ATP-binding protein identified by FeGenie (Supplementary Data Sheet S5). Additionally, FeGenie identified a locus with three genes, two of which are predicted to be involved in iron transport and one in gene regulation. The identified gene regulator, related to diphtheria toxin regulatory protein (DtxR), encodes TroR, which is activated by Mn^2+^ instead of Fe^2+^ (Posey et al., 1999). Likewise, the identified putative iron transport genes in that operon may be involved in the transport of Mn^2+^, rather than Fe^2+^, reflecting a strategy to circumvent iron limitation imposed by the host environment (Posey et al., 1999).

After validating FeGenie against isolate genomes, we utilized FeGenie to examine the iron-related genes and gene neighborhoods in environmental metagenomes, human oral biofilm isolates, and members of the CPR.

### Case Study: Iron Redox and Acquisition in Diverse Environmental Metagenomes

**FIGURE 5:**
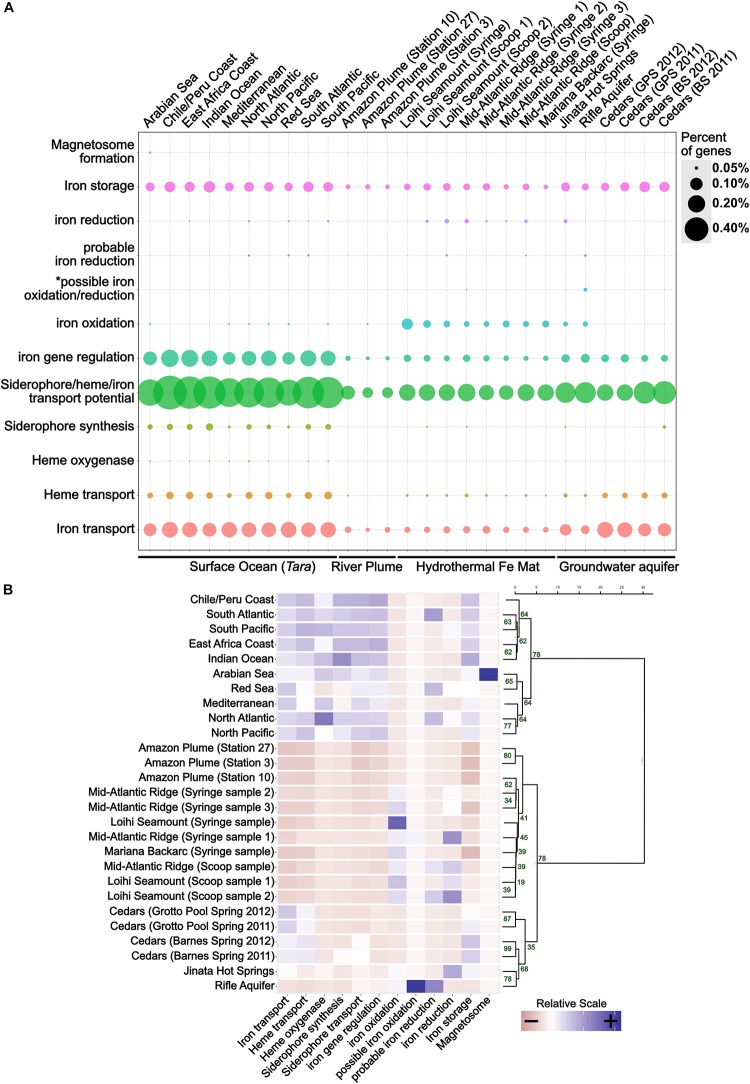
**(A)** Dot plot showing the distribution of iron genes on 27 metagenomes and **(B)** a scaled heatmap with accompanying dendrogram that represents hierarchical clustering of metagenome datasets based on identified iron genes. The dot plot shows the relative abundance of iron genes across 27 metagenomes. The size of each dot reflects the number of genes identified for each category, normalized to the number of protein-coding genes predicted within each metagenome. To generate the dendrogram, Ward’s method for hierarchical clustering was used, along with the Euclidian distance metric. The heatmap was created from a scaled version of FeGenie’s matrix output, which summarizes the amount of iron genes for each category present in each metagenome assembly. GPS, Grotto Pool Spring; BS, Barnes Spring. *This category is reserved for genes related to the mtoAB/pioAB gene family.

FeGenie was used to analyze 27 metagenomic datasets, representing a broad range of environments, including hydrothermal vent iron mats, a river plume, the open ocean, hot springs, and a serpentinite-hosted ecosystem (see section “Materials and Methods” and Table 2 for site descriptions). Generally, FeGenie’s analysis indicate that there are discernable differences in iron maintenance and metabolism strategies based on locale, likely due to differential iron availability and general redox conditions (Figure 5A and Supplementary Data Sheets S6, S7). For example, where iron oxidation and reduction gene counts are high, there appears to be fewer genes for iron acquisition. As expected, the genetic potential for iron acquisition and storage appears to be more important in environments where microorganisms are more likely to encounter iron limitations (Crosa, 1989; Andrews, 1998). This is supported by hierarchical clustering of the iron gene abundances across analyzed metagenomes (Figure 5B), an optional step in FeGenie’s pipeline. This offers support for FeGenie’s ability to provide meaningful insights into the iron-related genomic potential in environmental metagenomic datasets.

FeGenie demonstrates the potential for iron oxidation and reduction in environments that are rich in reduced iron, including the Rifle Aquifer (Jewell et al., 2016), Jinata Hot Springs (Ward, 2017; Ward et al., 2019), and iron mats found at the Loihi Seamount, Mid-Atlantic Ridge, and Mariana Backarc hydrothermal vent (McAllister et al., 2019). FeGenie also demonstrates the potential for these metabolisms to occur in other environments, including the Amazon river plume (Satinsky et al., 2017) and in the open ocean (Tully et al., 2018) (Figure 5A). While *cyc2* appears to be the most widely distributed gene that is associated with iron oxidation, other putative iron oxidases are also identified (e.g., sulfocyanin, *mtoAB*, *foxE*). Iron reduction is predicted from the occurrence of homologs to *mtrCAB*, as well as various porin-cytochrome operons homologous to those encoded by *Geobacter* and *Desulfovibrio* species. In addition, we identified homologs to the cytochrome OmcS from *Geobacter sulfurreducens*, thought to be involved in long-distance extracellular electron transfer (Wang et al., 2019), in Loihi iron mats and the open ocean. The presence of significant iron reduction in the open ocean water column is not expected due to generally low iron concentrations. However, as previously suggested by Chiu et al. (2017), niche-specific strategies, such as association with particulate matter or flocs, may take place in the iron-deplete water column and host microbially mediated iron cycling.

While iron oxidation and reduction are predicted in a range of environmental samples analyzed, the greatest number of iron redox genes are predicted in iron-rich ecosystems. Genes associated with dissimilatory iron reduction often coincide with those for iron oxidation. Exceptions to this include the upper centimeters (i.e., syringe samples) of iron mats from Loihi and the Mariana Backarc (McAllister et al., 2019); these samples encode many genes for iron oxidation but have no genes linked exclusively to iron reduction. This may indicate that (1) iron reducers form a non-detectable fraction of the community in those samples, (2) that the geochemical regimes present there do not favor dissimilatory iron reduction, or (3) that there are other, currently unknown, mechanisms for iron reduction occurring. For example, the surficial iron mat sample from the Loihi Seamount appears to have the highest number of genes related to iron oxidation, and none related to iron reduction; it also happens to be the sample dominated by the iron-oxidizing *Zetaproteobacteria* at 96% relative abundance (McAllister et al., 2019). Nonetheless, the predicted occurrence of iron reduction in most (7 out of 10) of the iron oxidizer-dominated ecosystems indicates potential interdependence, or even syntrophic interactions, between iron-oxidizing and iron-reducing microorganisms (Emerson, 2009).

Metagenomes from the Cedars (Suzuki et al., 2017), a hyperalkaline terrestrial serpentinite-hosted site, encodes a diversity of iron acquisition genes, similar to that observed in the open ocean, suggesting potential iron-limiting conditions. Accordingly, we did not detect any genes associated with iron reduction or oxidation. However, Gibbs energy calculations suggest that iron oxidation and reduction are both feasible metabolisms in serpentinite-hosted systems (Cardace et al., 2015), and electrochemical enrichment of a magnetite-reducer (Rowe et al., 2017) indicates that dissimilatory iron reduction may be occurring within the rare biosphere, biofilms on surfaces of iron-bearing minerals, or iron-containing flocs.

Genes potentially involved in magnetosome formation (*mam*) are present in only one of the 27 metagenomes analyzed: Arabian Sea surface waters (Tully et al., 2018). The one potential magnetosome-related operon from the Arabian Sea encodes six of the ten *mam* markers used (*mamMOPAQB*). FeGenie strictly reports potential homologs to the *mam* operon genes if the operon is at least 50% complete. Thus, the general lack of magnetosome formation in the other metagenomes could be a result of FeGenie’s strict rules. Alternatively, the microbial communities represented by these metagenomes either (1) do not have magnetotactic microorganisms present at a detectable level or (2) magnetotactic microorganisms present within these communities utilize an unknown strategy for magnetosome formation and/or magnetotaxis.

### Case Study: Iron Acquisition by Bacteria Living in the Human Oral Biofilm

The microbial capability to uptake iron is critical to understanding human oral infections (Wang R.K. et al., 2012). This is because host iron-binding proteins, such as transferrin, lactoferrin, hemoglobin, and ferritin, maintain an environment of low free iron concentrations (estimated 10^–18^ M free iron in living tissues; Weinberg, 1978), inhibiting bacterial growth (Mukherjee, 1985). Here, we used FeGenie to analyze four representative strains from the human oral biofilm community: *Aggregatibacter actinomycetemcomitans* Y4, *Capnocytophaga ochracea* DSM 7271, *Porphyromonas gingivalis* W83, and *Streptococcus mutans* UA159. Given that these four strains are members of the human oral biofilm (Welch et al., 2016), their iron acquisition systems may be tailored toward the specific strategies needed to survive in the human oral biofilm. Three of these isolates (all except *P. gingivalis*) show generally high numbers of genes involved in iron transport (Figure 4 and Supplementary Data Sheets S4, S5). *A. actinomycetemcomitans* and *P. gingivalis* have potential genes for heme transport, in line with a previous report of *P. gingivalis* being incapable of synthesizing heme, requiring exogenous iron addition for survival (Roper et al., 2000). *A. actinomycetemcomitans*, *P. gingivalis*, and *Streptococcus mutans* also show high genetic potential for siderophore uptake but have no genes implicated in siderophore synthesis. This suggests that if they do uptake siderophores, they may do so as “cheaters” (bacteria that uptake siderophores produced by other organisms) (Hibbing et al., 2010). In contrast, *C. ochracea* encodes both siderophore uptake and synthesis genes. No genes associated with dissimilatory iron reduction or oxidation were detected in any of the oral biofilm isolates.

### Case Study: Iron-Related Genes Encoded Within the Candidate Phyla Radiation and Other Candidate Taxa

FeGenie was used to identify the iron-related genes encoded by members of the Candidate Phyla Radiation (CPR) and other candidate taxa. These candidate taxa have been identified by metagenome or single-cell genome assemblies, but the microorganism has yet to be cultivated. Many of the CPR genomes have previously been reconstructed from a metagenome from the Rifle aquifer (Anantharaman et al., 2016) and are largely unexplored with respect to phenotype and role in the environment (Brown et al., 2015). Nonetheless, CPR members are defined by relatively small genomes and very limited metabolic capacity, suggesting that symbiotic lifestyles are likely prevalent among these phyla (Danczak et al., 2017). While we present results for only a select set of 17 candidate taxa (Supplementary Data Sheets S8, S9), all publicly-available genomes from the CPR and other candidate strains were analyzed (Supplementary Data Sheets S10, S11). The 17 selected genomes were chosen to demonstrate differences within these genomes with regard to genomic potential for iron acquisition, storage, and redox-cycling. The candidate strains presented here include members of the candidate phyla OP9 (Caldatribacterium), as well as “*Candidatus* Rokubacteria,” “*Candidatus* Nealsonbacteria,” “*Candidatus* Zixibacteria,” and the novel Archaeal phylum AR4.

**FIGURE 6:**
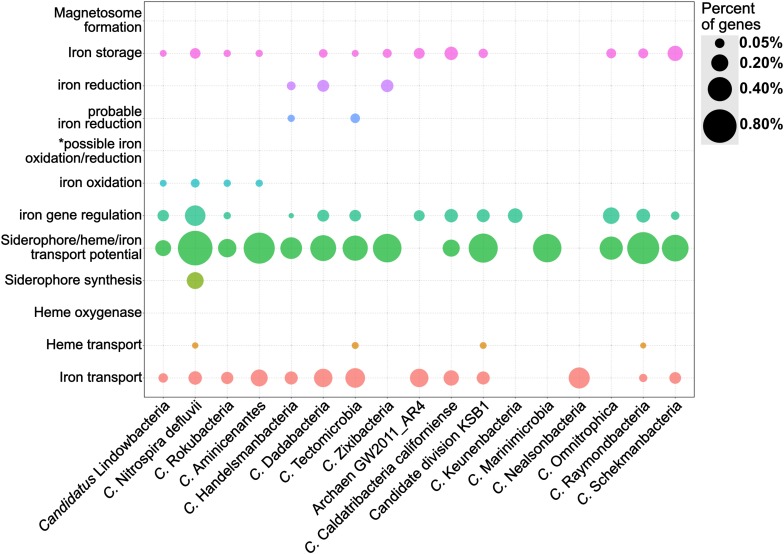
Dot plot showing the relative abundance of different iron gene categories in 17 genomes from the Candidate Phyla Radiation and other candidate taxa. The genomes were obtained from the NCBI RefSeq and GenBank databases and analyzed by FeGenie. *This category is reserved for genes related to the MtoAB/PioAB gene family.

Genes for siderophore synthesis were detected in only one of the candidate strains analyzed (Figure 6), while potential for siderophore transport is found in nearly all of the genomes. Gene candidates for heme transport genes, specifically *hmuV* and *hmuY*, were found in 4 of the 17 candidate strains analyzed: ‘*Candidatus* Raymondbacteria,’ ‘*Candidatus* Tectomicrobia,’ ‘*Candidatus* Nitrospira defluvii,’ and the genome from candidate division KSB1. Out of the 17 candidate strains analyzed, none were found to encode genes associated with heme oxygenases. Interestingly, some CPR genomes, such as “*Candidatus* Nealsonbacteria,” do not seem to encode any genes associated with iron maintenance or metabolism, with the exception of some putative iron transporters. One possible reason for this is that these microorganisms, whose genomes are considerably smaller than typical free-living bacteria, are obligate symbionts (Hug et al., 2016) and may be obtaining iron from their host or using the host’s cellular machinery for iron acquisition and utilization. Alternatively, these understudied phyla may be utilizing, thus far, undiscovered mechanisms for iron metabolism, and the genetic underpinnings of these mechanisms may not bear any homology to the HMMs included in FeGenie’s database.

*‘Candidatus* Lindowbacteria,’ *‘Candidatus* Rokubacteria,’ *‘Candidatus* Aminicenantes,’ *‘Candidatus* Handelsmanbacteria,’ and *‘Candidatus* Nitrospira defluvii’ show genetic potential for iron oxidation *via* homologs to either *cyc2* or sulfocyanin genes. ‘*Candidatus* Nitrospira defluvii,’ a close relative of the iron-oxidizing *Leptospirillum* (Lücker et al., 2010) also encodes *aclAB* and, thus, may be capable of carbon fixation via the reverse tricarboxylic acid cycle (rTCA). While this metagenome-assembled genome was previously reported as a potential nitrite-oxidizer (Lücker et al., 2010), here we report that it could potentially contribute to primary production using energy generated from iron oxidation. Within the genome of ‘*Candidatus* Tectomicrobia,’ FeGenie identified homologs to *mtrAB*. These iron reduction-related genes have not been previously reported in this candidate phylum (Wilson et al., 2014), demonstrating FeGenie’s ability to help identify biological processes not previously identified in other reports. *‘Candidatus* Zixibacteria,’ *‘Candidatus* Tectomicrobia,’ *‘Candidatus* Dadabacteria,’ and *‘Candidatus* Handelsmanbacteria’ also encode genes implicated in iron reduction via porin-cytochrome operons that share homology with those encoded by iron-reducing *Geobacter* spp. Taken together, these results suggest a potential role in iron cycling for some of the CPR members and other candidate taxa. Future culture-dependent, physiological work is needed to confirm this potential.

## Conclusion

Here, we describe a new HMM database of iron-related genes and a bioinformatics tool, FeGenie, that utilizes this database to analyze genomes and metagenomes. We validated this tool against a select set of 28 isolate genomes and demonstrate that FeGenie accurately detects genes related to iron oxidation/reduction, magnetosome formation, iron regulation, iron transport, siderophore synthesis, and iron storage. Analysis of 27 environmental metagenomes using FeGenie further validated this tool, revealed differences in iron maintenance and potential metabolic strategies across diverse ecosystems, and demonstrates that FeGenie can provide useful insights into the iron gene inventories across habitats. We also used FeGenie to provide insights into the iron metabolisms of 17 of the recently discovered CPR microorganisms and other candidate taxa, and revealed genetic potential not identified in previous reports. FeGenie will be continuously updated with new versions as new iron-related genes are discovered.

## Data Availability Statement

The datasets generated for this study can be found in the following GitHub repository: https://github.com/Arkadiy-Garber/FeGenie.

## Author Contributions

AG, NM, RB, and CC contributed to creating the HMM database. AG programed FeGenie. NM developed the concept. SM collected and processed metagenomic samples from the Loihi Seamount, Mid-Atlantic Ridge, and Mariana Backarc. AG, NM, AO, SM, CC, RB, and KN wrote the manuscript.

## Conflict of Interest

The authors declare that the research was conducted in the absence of any commercial or financial relationships that could be construed as a potential conflict of interest.

## Footnotes

1 https://conda.io/projects/conda/en/latest/

2 https://conda.io/projects/conda/en/latest/

3 https://github.com/Arkadiy-Garber/FeGenie/wiki/Installation

4 http://dx.doi.org/10.6084/m9.figshare.5188273

## Supplementary Material

FIGURE S1 Maximum-likelihood phylogenetic tree of six MtrA homologs used to build the MtrA HMM and six “MtoA” homologs used the build the MtoA HMM. This tree represents sequences only from confirmed iron oxidizers and iron reducers. It is important to note that, as far as we currently know, physiological evidence for iron oxidation or reduction capacity only exists for MtrA/MtoA homologs encoded by *Rhodopseudomonas palustris* TIE-1, *Sideroxydans lithotrophicus* ES-1, and *Shewanella oneidensis* MR-1.

TABLE S1 List of iron-related genes identified by FeGenie.

TABLE S2 Acquisition of representative genomes. Genome sequences of model microorganisms were downloaded from the NCBI RefSeq database on November 4, 2017 (Pruitt et al., 2007). Additional genome sequences for candidate taxa were obtained using the NCBI accession IDs found in Hug et al. (2016).

TABLE S3 FeGenie’s rules for identifying dissimilatory iron oxidation and reduction. These rules are derived from the genomic co-occurrence, operon structures, and phylogeny of genes previously implicated in these metabolisms.

TABLE S4 AntiSMASH results of the *Bacillus anthracis* genome. Cluster 17 and 19 are siderophore operons, where Cluster 17 is the operon that produces petrobactin and Cluster 19 is a siderophore(anthrabactin) with 46% of genes similar to the bacillibactin gene cluster.

DATA SHEET S1 Legend for HMM name to genes included in model.

DATA SHEET S2 FeGenie output for RefSeq representatives in heatmap-formatted table.

DATA SHEET S3 FeGenie output for RefSeq representatives in long-format summary.

DATA SHEET S4 FeGenie output for isolate genomes in long-format summary.

DATA SHEET S5 FeGenie output for isolate genomes in heatmap-formatted table.

DATA SHEET S6 FeGenie output for metagenomes in long-format summary.

DATA SHEET S7 FeGenie output for metagenomes in heatmap-formatted table.

DATA SHEET S8 FeGenie output for CPR and candidate strain genomes in long-format summary.

DATA SHEET S9 FeGenie output for CPR and candidate strain genomes in heatmap-formatted table.

DATA SHEET S10 FeGenie output for all CPR and candidate strain genomes in long-format summary.

DATA SHEET S11 FeGenie output for all CPR and candidate strain genomes in heatmap-formatted table.
