# Supplementary material for: FeGenie: A Comprehensive Tool for the Identification of Iron Genes and Iron Gene Neighborhoods in Genome and Metagenome Assemblies
Source: Front Microbiol. 2020 Jan 31;11:37. doi: 10.3389/fmicb.2020.00037 (PMC7005843; doi:10.3389/fmicb.2020.00037)
Supplement: FIGURE S1 — Maximum-likelihood phylogenetic tree of six MtrA homologs used to build the MtrA HMM and six “MtoA” homologs used the build the MtoA HMM. This tree represents sequences only from confirmed iron oxidizers and iron reducers. It is important to note that, as far as we currently know, physiological evidence for iron oxidation or reduction capacity only exists for MtrA/MtoA homologs encoded by Rhodopseudomonas palustris TIE-1, Sideroxydans lithotrophicus ES-1, and Shewanella oneidensis MR-1. [file Image_1.pdf]

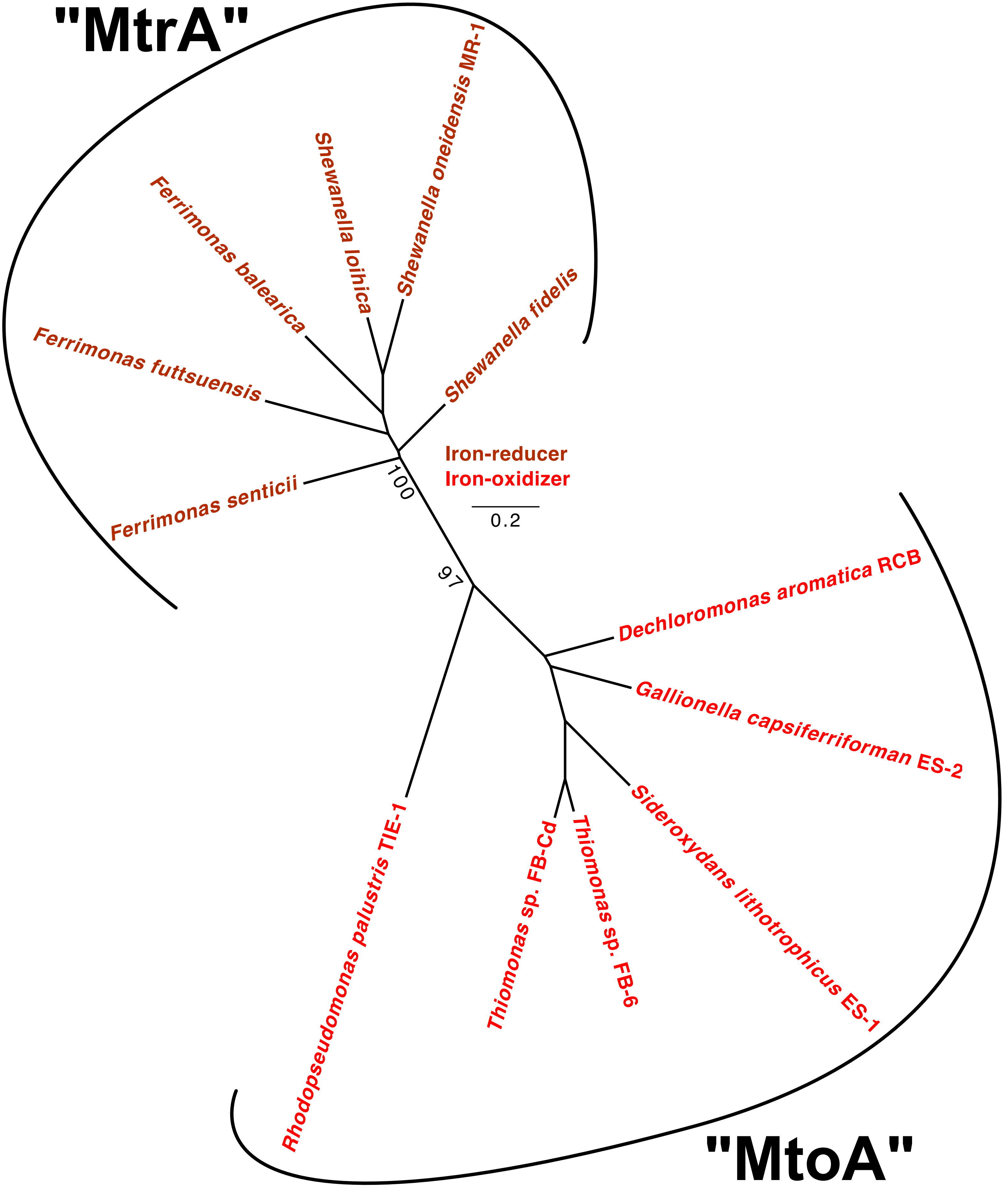

Supplementary Figure 1: Maximum-likelihood phylogenetic tree of six MtrA homologs used to build the the MtrA HMM and six "MtoA" homologs used the build the MtoA HMM. This tree represents sequences only from confirmed iron oxidizers and iron reducers. It is important to note that, as far as we currently know, physiological evidence for iron oxidation or reduction capacity only exists for MtrA/MtoA homologs encoded by *Rhodopseudomonas palustris* TIE-1, *Sideroxydans lithotrophicus* ES-1, and *Shewanella oneidensis* MR-1.
